# Motor output variability, deafferentation, and putative deficits in kinesthetic reafference in Parkinson’s disease

**DOI:** 10.3389/fnhum.2014.00823

**Published:** 2014-10-21

**Authors:** Elizabeth B. Torres, Jonathan Cole, Howard Poizner

**Affiliations:** ^1^Sensory Motor Integration Laboratory and Department of Psychology, Department of Computer Science and Rutgers University Center for Cognitive Science, Rutgers University-New BrunswickNew Brunswick, NJ, USA; ^2^Neurology, Poole Hospital NHS FoundationPoole, UK; ^3^Institute for Neural Computation, University of California at San DiegoSan Diego, CA, USA

**Keywords:** motor output variability, kinesthetic reafference, Parkinson’s disease, stochastic analyses, noise

## Abstract

Parkinson’s disease (PD) is a neurodegenerative disorder defined by motor impairments that include rigidity, systemic slowdown of movement (bradykinesia), postural problems, and tremor. While the progressive decline in motor output functions is well documented, less understood are impairments linked to the continuous kinesthetic sensation emerging from the flow of motions. There is growing evidence in recent years that kinesthetic problems are also part of the symptoms of PD, but objective methods to readily quantify continuously unfolding motions across different contexts have been lacking. Here we present evidence from a deafferented subject (IW) and a new statistical platform that enables new analyses of motor output variability measured as a continuous flow of kinesthetic reafferent input. Systematic increasing similarities between the patterns of motor output variability in IW and the participants with increasing degrees of PD severity suggest potential deficits in kinesthetic sensing in PD. We propose that these deficits may result from persistent, noisy, and random motor patterns as the disorder progresses. The stochastic signatures from the unfolding motions revealed levels of noise in the motor output fluctuations of these patients bound to decrease the kinesthetic signal’s bandwidth. The results are interpreted in light of the concept of kinesthetic reafference ( Von Holst and Mittelstaedt, 1950). In this context, noisy motor output variability from voluntary movements in PD leads to a returning stream of noisy afference caused, in turn, by those faulty movements themselves. Faulty efferent output re-enters the CNS as corrupted sensory motor input. We find here that severity level in PD leads to the persistence of such patterns, thus bringing the statistical signatures of the subjects with PD systematically closer to those of the subject without proprioception.

## INTRODUCTION

There is growing evidence that problems with kinesthetic sensitivity are common in Parkinson’s disease (PD; Maschke et al., 2006; Nolano et al., 2008; Carpenter and Bloem, 2011; Tan et al., 2011). Such problems already manifest at very early stages of the disorder, when motor problems are still very mild (Konczak et al., 2007, 2008), suggesting that kinesthetic dysfunction may precede the observable motor impairments in more severe stages of the disorder (Konczak et al., 2009). Subtle sensory issues are detectable in the lab settings with appropriate instrumentation. Yet, they are often missed by observational inventories that depend on human inference and verbal reports. There is, however, evidence from early reports suggesting that over 40% of PD patients suffer from such problems which neurological examinations might miss (Koller, 1984).

Clinical rating methods such as the UPDRS (Goetz et al., 2008; Berg et al., 2014) could be complemented with detailed kinematic analyses to detect, within the fluctuations of motor output variability, statistical signatures of noisy kinesthetic motion input during early stages of the disorder. Motor output variability, continuously flowing as we move around, can be thought of as one of the sources of kinesthetic feedback in light of Von Holst’s concept of movement reafference (Von Holst and Mittelstaedt, 1950). Motor output variability is readily measurable, partly because peripheral nerve signals are naturally amplified by the muscles (Kuiken et al., 2004, 2007). Such sources of variability are known to contain a blend of signal and noise informative of control strategies of the nervous systems (Scholz et al., 2000; Latash et al., 2002; Faisal et al., 2008). They are also informative of anomalies in kinematic variability that subjects with PD manifest, such as failure to balance goal-directed and spontaneous movements as they unfold (Torres et al., 2011; Yanovich et al., 2013).

In this paper we hypothesize that *deficiencies in movement sensing may emerge, even at a mild stage of PD, when statistically noisy and random motor output variability persists over years*. We propose that a type of subtle “virtual deafferentation” may emerge from persistent random and noisy movement sensing. Over time, corrupted motor output variability may lead to a sort of kinesthetic sensory atrophy, over reliance on external sensory input and poor integration of somatosensory and motor signals. The continuous flow of motor output variability is a source of kinesthetic sensory guidance to the central nervous system, part of a continuous returning stream caused by our own movements as the CNS volitionally controls them. This form of input normally bears statistical information confirming estimation and prediction of the sensory consequences of our actions to gain confirmation in favor or against our estimations (Torres, 2013b). If the bandwidth of the peripheral signal-to-noise ratio is persistently narrow and corrupted, this may impact centrally driven processes and force the system to compensate by relying on external sensory input.

This sensory uncertainty may be one reason for the findings, from several researchers, of over reliance in visual guidance in PD for reaching (Flash et al., 1992; Klockgether and Dichgans, 1994; Adamovich et al., 2001; Torres et al., 2011) and grasping (Jackson et al., 1995; Schettino et al., 2006; Muratori et al., 2008; Lukos et al., 2013) movements. In particular, we had previously detected over reliance on visual cues for movement guidance in a subset of patients in this PD cohort (Adamovich et al., 2001; Torres et al., 2011). Using the variability in the endpoint errors of reaching movements we had found that in the absence of visual guidance, when relying on proprioception and a memory of the target, these subjects had very large end point position and arm postural errors (Torres et al., 2011). Yet, with visual guidance of their moving finger they improved performance toward typical regimes.

Here we assess the micro-structure of movement variability in parameters of the unfolding hand motions of a heterogeneous PD cohort, in relation to subject IW, who lacks proprioception (Cole, 1995). We ask whether from trial to trial, as the movements continuously unfold, the absence or presence of visual guidance has similar impact on the stochastic signatures of motor output variability of the subject without proprioception as it does on the subjects with PD. We further examine the degree of severity of the PD subjects with respect to the noise levels in their motor output variability.

## MATERIALS AND METHODS

### PARTICIPANTS

We asked 26 patients with a diagnosis of PD to perform the task. Two main groups were examined: one with mild PD previously examined but addressing different issues, and another examined more recently. The arm movement structure in three-dimensional space was similar for all patients: point forward to the location of a target without touching it and spontaneously retract the hand to rest within a continuous loop. In all cases the subject just had to point to the target without making contact with it, but in group one the target was presented by a robot whereas in group 2 the target was presented on a computer screen. In both groups of patients, subjects either had not taken their anti-parkinsonian medication for at least 12 h before testing or were tested at the end of dose when the medications were losing effectiveness (Langston, 1991; Defer et al., 1999). See also Adamovich et al. (2001) for additional details on the mild PD patients.

We also tested eight age-matched elderly healthy subjects (three females and five males) to perform visually and memory-guided pointing motions in three dimensions. In addition to the eight age-matched elderly controls, we also examined the forward and back 3D-pointing motions of 25 right handed young controls (ages ranging between 23 and 29 years old, 7 females and 18 males).

**FIGURE 1:**
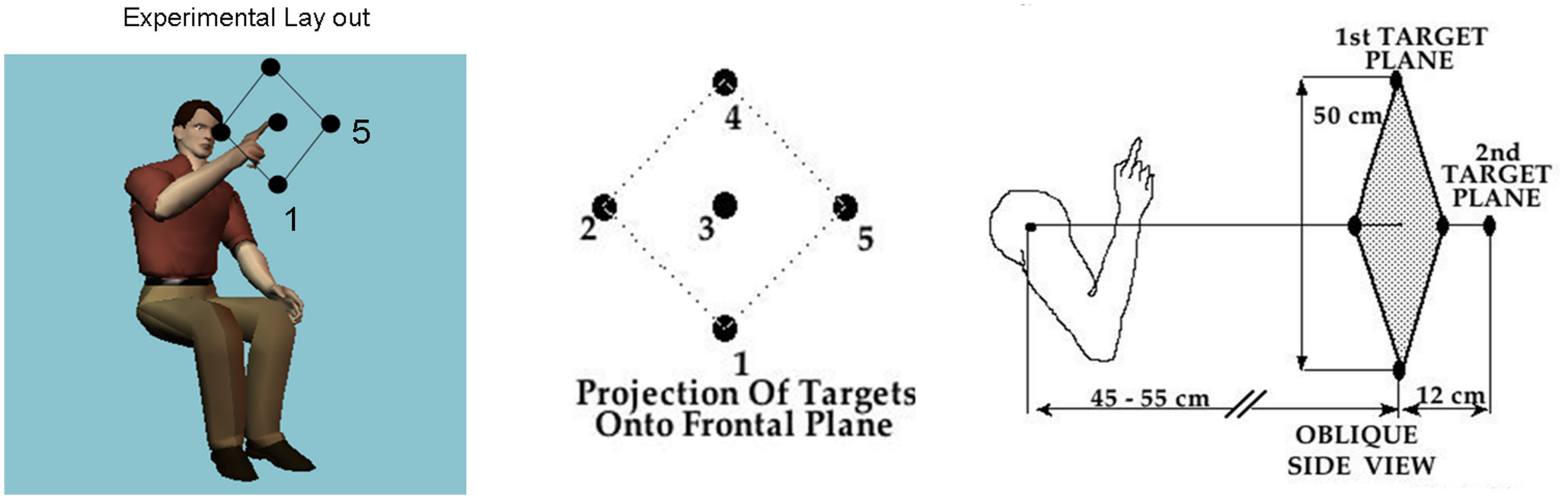
**Basic task.** Schematic diagram shows the participant seated and pointing at the central target. The projection of all 5 targets on the frontal plane is shown next to the arm in the initial position in relation to the five targets in a slightly rotated side view. Participants performed visually and memory-guided reaches toward all targets and backward to rest under these different conditions.

In all cases the pointing movements were in three dimensions along a continuous loop to one of five targets (shown in **Figure 1**) and back to rest. Their outward reach had various conditions, see below, but there were no instructions for the return motion. Additional clinical descriptions and medication of the PD patients are presented in **Tables 1 and 2**.

**Table 1:** Mild PD group.

Subject	Sex	Age	Stage^1^	UPDRS^2^	Symptoms (years)^3^	Medicines
1	M	73	2.0	24.7	25	L,Per, Tri
2	M	75	2.5	a	16	E, LS, Pro
3	M	74	2.5	47.8	10	Be, L, S
4	F	79	3.0	15.8	4	A, L, Pra, S
5	M	75	2.5	27.0	8	Bu, C, Lu, S
6	M	77	3	28.5	9	none
7	M	58	2.0	21.5	8	L, LS, To, Pe
8	M	72	3	43.5	5	Pe, S
9	M	58	2.5	30.1	4	L, S

Summary	1F/8M	71.2 ± 7.8	2.5 ± 0.4	29.8 ± 10.7	9.8 ± 6.7	

*Demographic and clinical features of nine PD patients tested in the “off” state (UPDRS unified PD rating scale; motor subscale. In some cases patients were studied on more than one day. UPDRS scores were then averaged. In some cases, the UPDRS examination was not done for all items. In this case, the score was normalized to a total possible score of 108. Anti-parkinsonian and related medications: A, amantidine; Be, benztropine; Bu, buspar; C, clonazepam; E, vitamine E; L, levodopa preparation regular release; LS, levodopa preparation, sustained release; Lu, ludiomil; No, notriptyline; Pe, pergolide; Pra, pramipexole; Pro, propanolol; S, selegiline; Sy, synthroid; T, trihexyphenidyl; To, tolcapone; ^a^UPDRS scores unavailable for this patient.*

*^1^Hoehn and Yahr stage.*

*^2^Maximum score 108.*

*^3^Refers to number of years since first remembered parkinsonian symptoms.*

**Table 2:** Advanced PD group.

Subject	Sex	Age	Stage^1^	UPDRS	Symptoms (years)^2^	Medicines
1*(DBS@47)	M	52	3	27	14	A; L; Pra
2*(DBS @48)	M	59	4	42	19	L
3*(DBS @70)	F	81	4	38	20	L; Pra
4	F	64	3	28	4	L
5	M	41	2	15	2	A; L
6	M	57	3	29	4	L; Pra
7	M	49	3	42	6	Ras; Rop; Tri
8	M	70	3	31	3	L
9	M	80	4	42	9	L
10	F	67	3	31	5	L; Ras
11	F	77	4	35	6	L; Pra
12	F	55	3	27	6	L; Ras; Rop
13	M	77	3	31	7	Don; L
14	M	72	2	21	8	L; Pra; Ras
15	M	54	4	42	9	L; Rop; Sel
16	M	60	3	35	10	A
17	F	69	2	18	12	L; Pra; Ras

Summary	6F/11M	63.7 ± 11.7	3.1 ± 0.7	31.4 ± 8.4	8.4 ± 5.2	

Demographic and clinical features of 17 PD patients tested in the “off” state (UPDRS unified PD rating scale; motor subscale. Antiparkinsonian medication as in **Table 1**; Ras, rasagiline; Rop, ropinirole.

*Deep Brain Stimulation at age.

*^1^Hoehn and Yahr stage.*

*^2^Refers to number of years since first remembered parkinsonian symptoms.*

**FIGURE 2:**
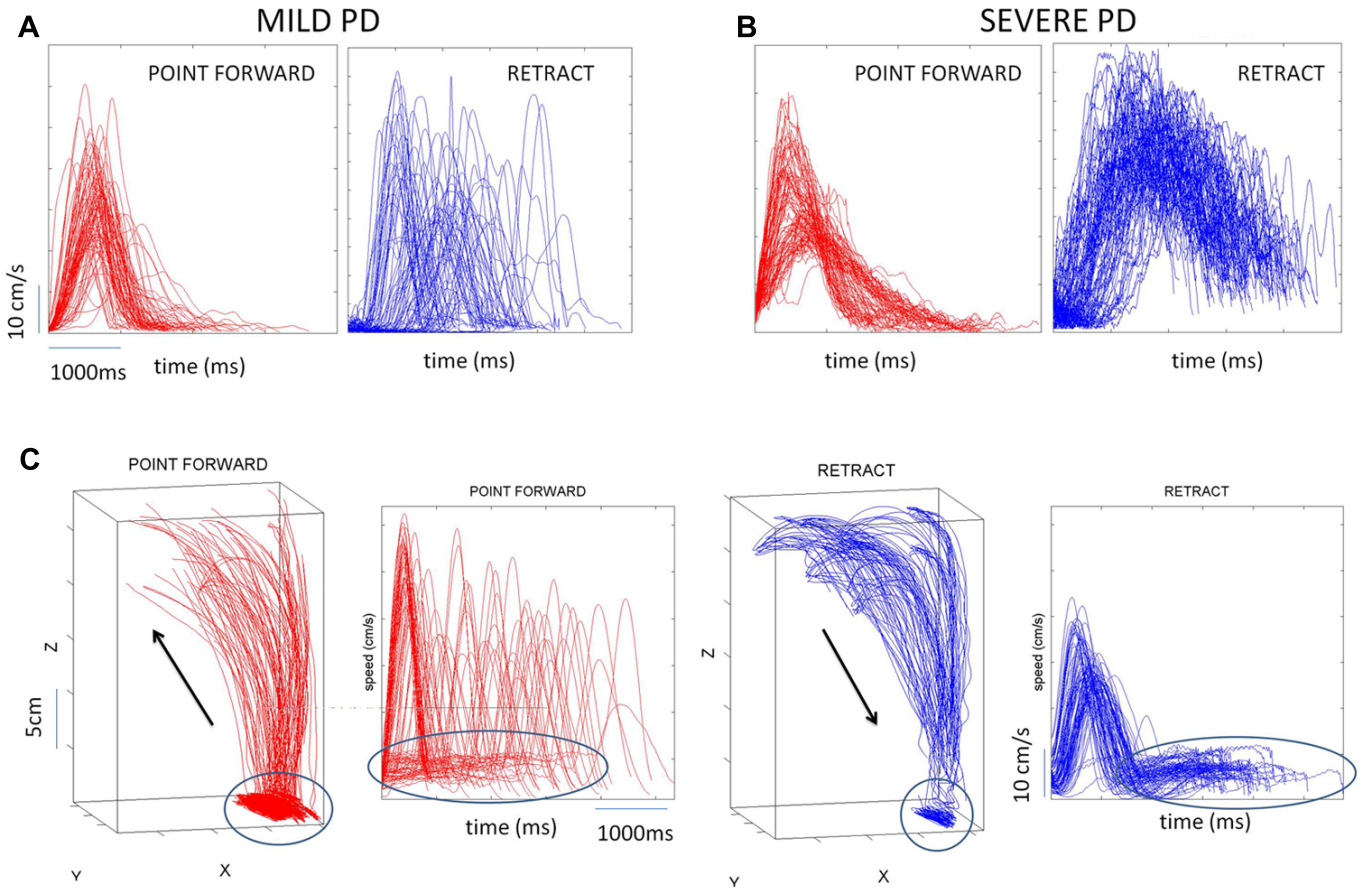
**Seed profiles of representative mild and severe Parkinson’s disease (PD) patients. (A)** Representative mild PD patient’s speed profiles. **(B)** Severe PD patient’s speed profiles. Notice the “jitter” in these profiles particularly on the spontaneous retraction, adding to the overall variability patterns. **(C)** Sample trajectories and speed profiles of another severe PD patient with problems initiating motions and resting tremor as well (both epochs circled).

Briefly, the first group of nine PD patients was composed of all mild to moderate in degree, Hoehn and Yahr stage 2–3. The other 17 PD patients were in Hoehn and Yahr stage 3–4. These patients’ main functional impairments likely were due to balance and gait problems, given that they could not walk independently. Unlike the first, this group showed prominent action tremor which was functionally impairing. Such features were not as noticeable in the traces of the trajectories of the mild group, but were very pronounced in those of the other group, particularly in the speed profiles, an issue that we will further explore in the paper. We will use the nomenclature of mild PD to refer to the first group and severe PD to refer to the second group. **Figure 2** plots hand traces and corresponding speed profiles forward to a target and retracting from it using data from a representative patient in each group.

The mean (SD) United PD Rating motor scores was 26 (5.3) for the mild PD group and 31 (8.4) for the severe group. All patients had clinically typical PD, as reviewed by at least one movement disorder specialist and their motor disabilities were responsive to anti-Parkinsonian medications. No patient in the mild group had any off-state action tremor or dyskinesia of more than minimal amplitude. In the severe group most patients had bi-lateral tremor. All subjects in the mild group were right handed (Oldfield, 1971) and reached with their right arm. In the severe PD group all but one patient were right handed and reached with their right arm.

Elderly controls were healthy individuals with no reported sensory-motor impairments. Young controls were healthy college students who participated in a similar pointing experiment at a later time (see more detailed explanation later on.).

### DESCRIPTION OF THE DEAFFERENTED SUBJECT

Subject IW was a 43 year-old deafferented man (at the time these data were collected) with a complete large fiber sensory neuronopathy. He lost proprioception at the age of 19 due to a viral infection (Cole, 1995, 1998; Cole and Paillard, 1995). He lost the senses of movement, position, pressure, and light touch from the neck down (C3 level), but retains pain and temperature sensation. Motor nerve function was not affected but initially he was unable to move in any controlled manner due to proprioceptive loss. Since then, IW has learned to use visual supervision and motor imagery to replace the prediction of sensory consequences with virtual, often imagined, feedback of where his limbs should be. There is also some evidence that he employs feedforward control of his motions (Balslev et al., 2007; Miall and Cole, 2007) in ways that are likely to differ from those used by people with intact motor reafference. Specifically, subject IW constantly makes deliberate plans for immediate actions. He reports reliance on visual supervision and motor imagery to plan ahead every motion. Most crucially, he has to attend to and think about all actions, whether postural ones such as sitting, or any movement. He reports that any reduction in cognition, for instance a head cold, forces him to go to bed. If he cannot think, he cannot move.

IW is left-handed and pointed with his left hand, while the control subjects are right-handed and pointed with their right hands. Since the positioning of the targets relative to the pointing arm was constant across groups, direct comparisons of IW’s patterns with those of the other groups could be made.

### RATIONALE FOR THE KINEMATIC PARAMETERS OF CHOICE

Measures of motor output variability often refer to the end point spread of the reach at the target. The literature of motor control is rich in computational modeling and experimental work (Harris and Wolpert, 1998; van Beers et al., 2002, 2004; van Beers, 2009) demonstrating that such sources of variability are relevant to the planning and execution stages of goal-directed reaching movements. Less explored however, has been the variability of such motions as the movements continuously unfold.

In a series of papers we have recently explored the velocity- and acceleration-dependent variability of natural, unconstrained, continuously unfolding movements in 3D. We have discovered that this type of variability is highly informative of the severity in neurological disorders. In particular, in PD patients the velocity- and acceleration-dependent variability have revealed noise and randomness in their signatures that can blindly predict the severity of the disorder (Torres, 2013a; Yanovich et al., 2013).

Despite their noisy patterns and the lack of balance between the deliberate forward and the spontaneous retracing segments of these continuous loops of pointing motions (Torres et al., 2011; Torres, 2013a; Yanovich et al., 2013), we had also found that subjects with mild PD had considerable gains in hand endpoint performance and arm-postural control when guiding their pointing movements by vision of their moving finger (Adamovich et al., 2001; Torres et al., 2011). Here we have the opportunity to compare this PD cohort to subject IW under manipulations of visual guidance. We seek to obtain new metrics of noise-to-signal ratios from the motor output variability indicative of the degree of PD severity. To this end we use a new statistical platform for individualized behavioral analysis (SPIBA; Torres and Jose, 2012) designed to determine individual signatures of the sensory-motor system and track their shifts within the timescale of the experimental session across manipulations in sensory guidance.

### DESCRIPTION OF PROCEDURES OR INVESTIGATIONS UNDERTAKEN

The subjects were seated with their dominant arm flexed at the elbow, forearm being semi-pronated and vertical such that the hand was on a sagittal plane that was about 10 cm to the right of the subject’s ear (**Figure 1**). The subjects faced a programmable robot arm (Hudson Robotics, CRS 255A) that presented targets in 3-D space. A small light-emitting diode was attached to the tip of the robot’s arm and served as the target. Two optoelectronic cameras (Northern Digital, Inc.,) were used to record positions of five infrared emitting diodes (IREDs) that were affixed to the following segments of the subject’s limb: the acromial process of the scapula (shoulder), the lateral epicondyle of the humerus (elbow), the ulnar styloid process (wrist), as well as on the nail of the index fingertip, and on the robot arm tip. The subjects were asked to fully extend their right forefinger and not to move it with respect to the wrist. 2-D coordinates of the IREDs were monitored by each camera. Data from both cameras were sampled at 100 Hz and stored as 2-D binary files. Then they were low-pass filtered using a Butterworth filter with a cut-off frequency of 8 Hz, and three-dimensional coordinates were reconstructed.

The robot randomly presented five targets in two planes (**Figure 1**). Four targets formed a diamond in a frontal (coronal) plane. The geometric center of this diamond was on a sagittal plane that was defined by the subjects’ right shoulder, but was approximately 43–48 cm in front of the right shoulder. The two diagonals of the diamond were about 50 cm long. The fifth target was located on a sagittal plane directly in front of the right shoulder, but approximately 12 cm further from the shoulder than the four target diamond. Exact distances from the shoulder were individualized for each subject by first positioning the furthermost (fifth) target at a distance approximately equal to the length of the subject’s arm with the subject’s fingers being clenched. This positioning of the subject relative to the target prevented the subject from having to fully extend the arm to reach any of the targets.

All subjects reached using their dominant arm. Their initial limb position, as mentioned above, was with their dominant arm flexed at the elbow, forearm being semi-pronated and vertical such that the hand was on a sagittal plane that was about 10 cm to the right of the subject’s right ear, for right handed subjects, 10 cm to the left of the left ear for left-handed subjects. The subjects attempted to “touch” the target with their right forefinger and returned their arms to their initial positions in one smooth movement without corrections. Short pauses at the target naturally occur and as such were allowed so as to not constrain the motions. However, deliberate error corrections at the target were discouraged. The reaches were performed under different conditions, with vision of the target but not the arm, having to memorize the target and reaching with or without vision of their finger as guidance. In all experimental conditions the robot arm held the target position for 1.5 s, during which time the subject was able to view the target. The experiment was conducted in a darkened room. Vision of the target was provided by an illuminated LED attached to the tip of the robot end-effector. Vision of the moving finger was provided in some sessions with an illuminated LED attached to the nail of the index finger.

All these conditions add variability to the reach. In previous publications we had separately examined these conditions in detail (Adamovich et al., 2001; Torres et al., 2010, 2011). However, for the purposes of this work, we examine the velocity-dependent variability of all the movements combined where some form of visual guidance was provided. We also examined the case where no visual guidance was provided and subjects had to rely on the memory of the target. In all cases we had at least (5 targets × 10 repetitions × 2 segments) trials.

### PD PATIENTS IN RELATION TO DEAFFERENTED IW

In all patients and deafferented subject we examined the trial-by-trial variability of velocity dependent parameters. These included the value of the peak velocity of each segment and the time when this peak value was attained. We examined their signatures of variability as the forward motion unfolded and also as the retracting motion back to rest took place under the different conditions.

In addition to the maximum speed value and timing of each segment we also quantified the frequency distributions of the values of additional local peaks (cm/s) present in the segments (forward and retracting) along with the frequency histograms of their inter local-peaks time intervals (ms). This quantification was motivated by the visible disparity between forward and back segments when comparing the traces from the motions of mild vs. severe PD patients (**Figures 2A vs. 2B**).

We used distributional analyses and estimation methods to examine the stochastic signatures of motor output variability of the patients with PD and normal controls (elderly and young) in relation to IW’s signatures.

### ADDITIONAL ANALYSES FOR DEAFFERENTED SUBJECT IW

IW’s motions were extensively studied both in the dark and also in a lit room. He received in some sessions visual guidance from the target (Target Vis) or from the LED attached to his pointing finger (Finger Vis). In other sessions he moved in complete darkness relying on the memory of the target or on his imagination (No Vis). Elderly controls and patients with PD also underwent these conditions in the darkened room. For the purposes of the comparison, we pooled all trials without visual feedback and all trials with visual guidance to compare the best and worst case scenarios for IW. He is seemingly “typical” under visual guidance (i.e., similar to controls) but the stochastic signatures of his movement output variability under such conditions had never been assessed before.

Other sessions manipulated IW’s movement dynamics asking for fast or slow speeds or by placing loads on his arm. These sessions enabled us to systematically track the effects of all these conditions on the signatures of IW. We examined subject IW and determined the best and the worst statistical regimes as a function of context and experimental conditions.

### CONTROLS

Lastly, the young controls performed the same three dimensional forward-and-back pointing task to a visual target as the other participants. As with the other participants, the young controls did not have to touch the target, just point to it. The experiments took place in a lit room and they had continuous visual feedback of the target. These were all optimal performance conditions in addition to their being young and healthy, at the peak of their performance. We used the young controls data as an anchor to establish the best possible statistical case scenario and to examine the normally aging group against the young group. We also used the subject without proprioception at the other end of this performance spectrum under visual guidance and dark conditions. These extreme cases (deafferented and young) enabled us to set extreme bounds from best to worst and helped us identify self-emerging systematic patterns across the heterogeneous PD cohort indicative of severity levels.

**FIGURE 3:**
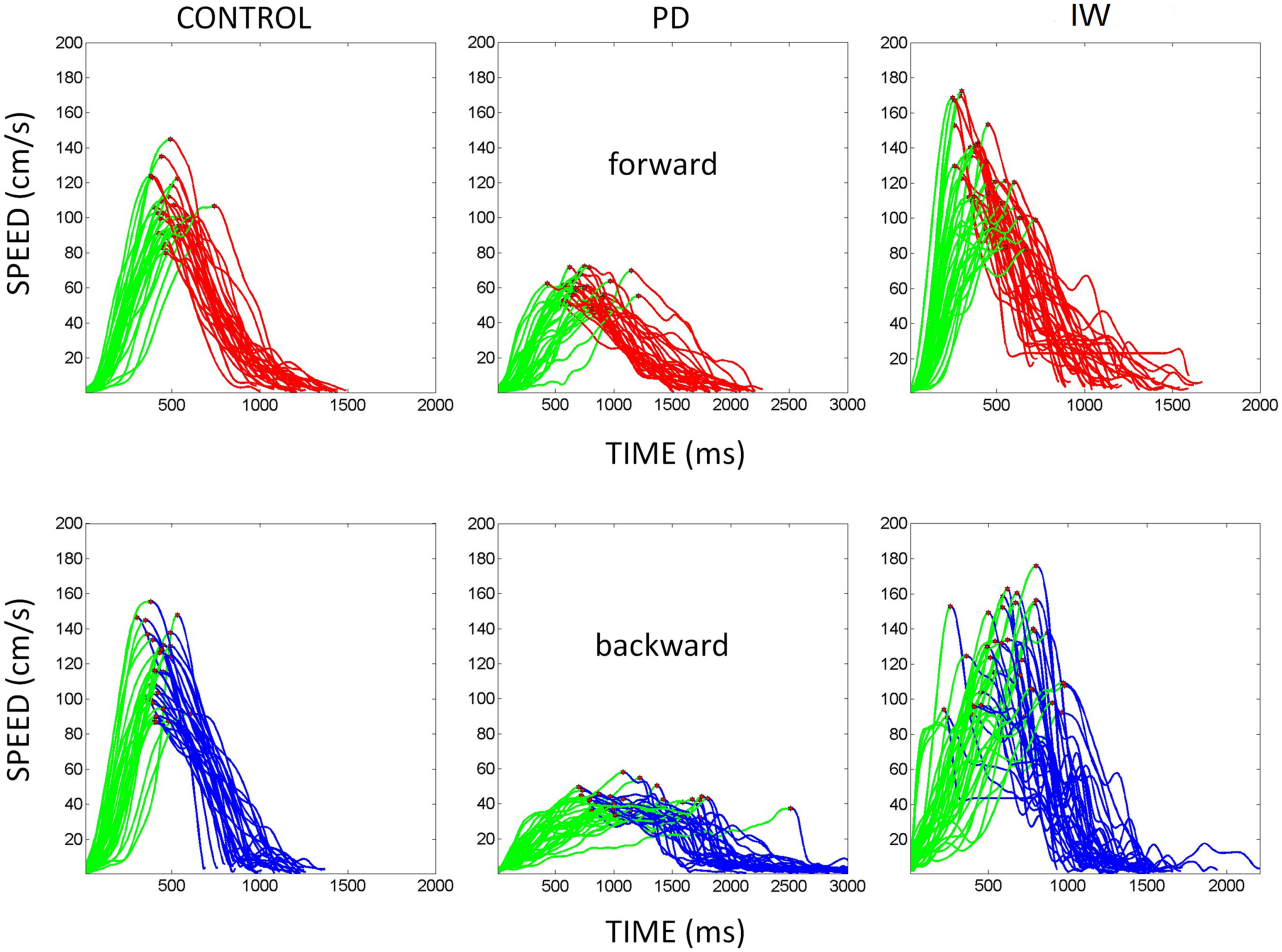
**Hand speed profiles.** Sample hand speed profiles from the forward and backward segments of the continuous reach. These are from different representative participants (normal age-matched control, PD patient, and IW). The peak velocity is marked in each trajectory segment. Also the distance traveled up to the peak velocity is marked in green for each of the forward and backward continuous reach segment.

Examples of the speed profiles from representative participants are shown in **Figure 3**. The various behavioral landmarks of interest are highlighted as well. These include the value of the peak velocity and the time to reach that value from the onset of the motion. We also highlight the initiation of the reach up to the peak in each case to contrast it with the deceleration phase of the movement.

### ETHICS

All procedures were undertaken with the understanding and written consent of each subject. The Rutgers University and the University of California, San Diego’s Institutional Review Board approved the study. The study conforms to The Code of Ethics of the World Medical Association (Declaration of Helsinki).

### STATISTICAL AND ANALYTICAL MEASURES

In the context of pointing movements with and without visual guidance, we explore the trial by trial velocity-dependent parameters as the movements continuously unfold. These parameters include the peak velocity and the time when this value is reached relative to the onset of movement. We examine these parameters in normalized form (later explained) to avoid possible confounds that could be introduced by anatomical differences across subjects.

The statistical platform that we use does not take averages of kinematic parameters to cast the results relative to normative population data. We do not assume that the continuous random process describing repeated pointing motions is Gaussian. Instead of conjecturing that the mean and variance parameters of the theoretical Gaussian probability distribution are good descriptors of this random process, we actually (empirically) estimate the family of probability distributions best describing the experimental data. Such estimation process is centered on the individual, rather than relative to assumed population statistics. Any statistical pattern or signature-clustering in the cohort itself emerges from the intrinsic stochastic properties of the data. Such patterns are based on the moment to moment variability of the kinematic parameters of interest for each individual in the cohort.

It is usually difficult to separate in the motor output variability the efferent from the afferent contributions to signal-noise flow. Since subject IW lacks movement proprioception, we use his results as an anchor to reference the statistical signatures of the PD subjects. In the absence of proprioception from movements, subject IW relies on vision to close the feedback loops. In lit conditions, IW relies on vision to control accuracy and when guided by vision his motions appear normal on average. He uses visual imagery and memory when no informative vision is available. How his movement variability unfolds from moment to moment is an unexplored question.

We compare the noise in the variability of the motor output from IW and that from the PD subjects under similar contexts. These patients have different UPDRS and motor scores (**Tables 1 and 2**). One question is whether the patients’ signatures approach those of IW in the dark or under visual guidance. To address this question we compare these PD subjects to IW performing reaches under similar dark and visually guided contexts. We also compare them to the healthy young participants as they optimally perform similar reaches under full visual guidance.

**FIGURE 4:**
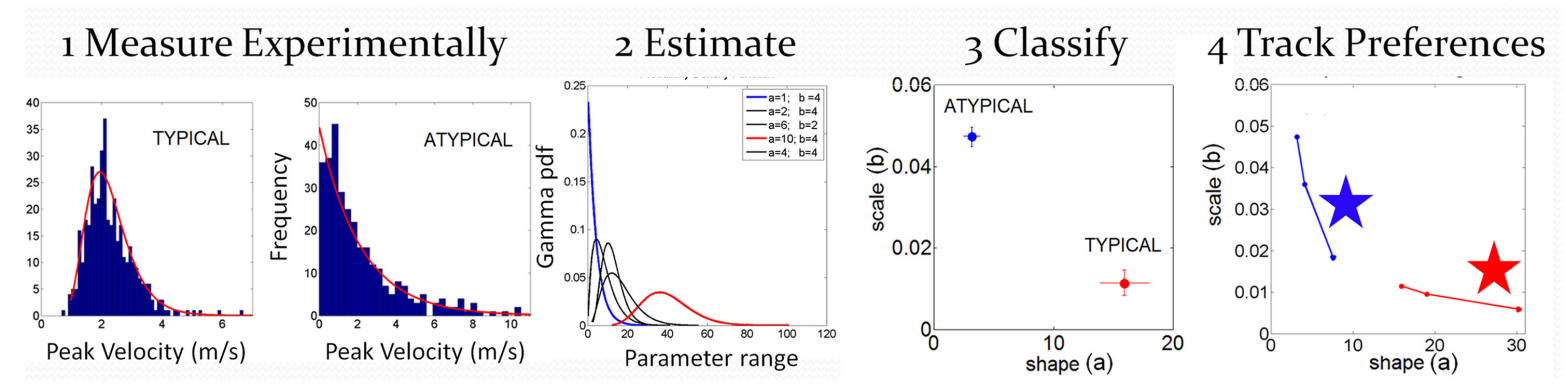
**Analytical methods: steps to convert from raw kinematics to estimated stochastic parameter trajectories so as to track their rates of change unique to each person.** Data is from hand pointing motion trajectories of human subjects using velocity dependent parameters.

The individualized statistical estimation procedure for each participant’s patterns of velocity-dependent motor output variability has three main layers displayed in **Figure 4**: (1) Acquiring the raw kinematics from the unconstrained continuous arm movements of the full forward and backward motion loop; (2) Estimating the stochastic signatures of the continuous reaching loops and mapping the participants on the plane of the Gamma probability distribution family (see below) relative to the stochastic signatures of IW at his worst and best contexts; (3) Generating various statistical indexes to further quantify individual features and to examine these in relation to self-emerging stochastic patterns in the cohort.

#### Raw kinematics

We examined the peak velocity (marked in **Figure 3** on the speed profiles of the hand trajectories) properly normalized to avoid allometric effects contributed by differences in anatomical length across subjects (Mosimann, 1970; Lleonart et al., 2000). The normalized peak velocity of the hand trajectory is the peak velocity value (m/s) divided by the sum of the trajectory’s peak velocity and its average speed (both in m/s) thus yielding a unitless quantity. If the averaged speed is low, the normalized peak velocity will be higher. In this sense, it is expected that in PD patients the normalized peak velocity may have higher values than that of controls (who move faster on average.)

Another parameter of interest is the time (ms) to reach the peak velocity. Here we obtained the percentage of such time out of the total trajectory duration. Lower values of this parameter indicate fast movement initiation.

The forward and back hand motion was performed as a continuous movement without corrections at the target. We examined the trial by trial variability of the parameters from both of these segments combined. We also examined the spread from each individual segment.

In addition to the maxima-dependent data (velocity value and timing) we examined the frequency distributions of *local* peaks in each segment along with their inter-time intervals. We aimed at unveiling random-noisy patterns that could further automatically distinguish systematic differences within the heterogeneous PD cohort. Action tremor patterns were visibly different in the retraction motions of these PD patients (**Figures 2A vs. 2B**). Could these differences be automatically quantified in their stochastic signatures?

#### Estimated stochastic signatures

To extract the stochastic signatures from the motor output variability (i.e., from the spread of these kinematics parameters over repetitions) we performed distributional analyses.

**FIGURE 5:**
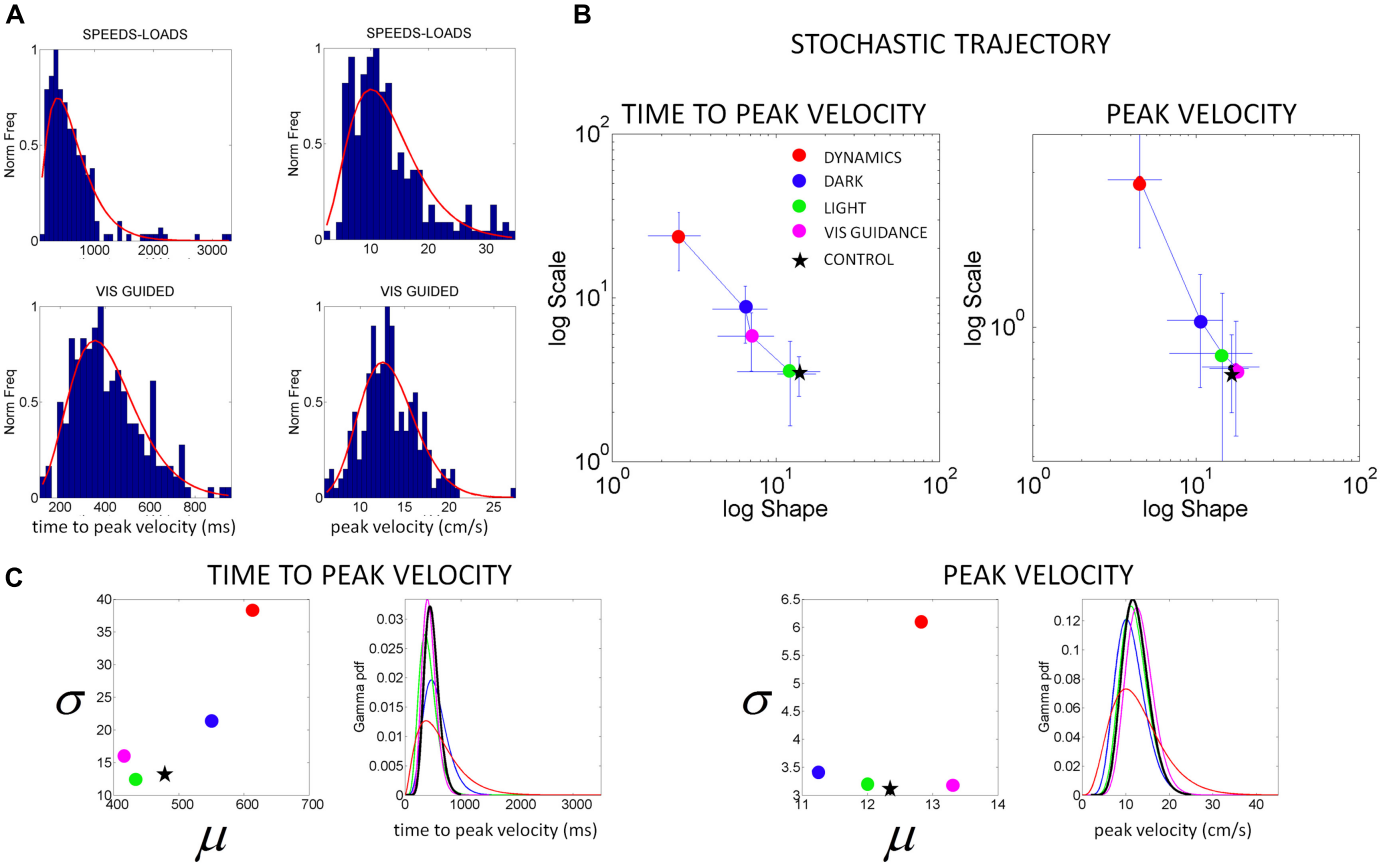
**Characterization of motor output variability in patient IW without proprioception for different reaching contexts. (A)** Frequency histograms of the time to reach the peak velocity (ms) and of the peak velocity value (cm/s) for 3D pointing motions to various target locations across different speeds and loads (top) and under visual guidance in the dark (lower). Red curve is the m.l.e. fit from the continuous Gamma family of probability distributions. **(B)** Stochastic trajectories of the kinematics parameters in **(A)** obtained within the time scale of an experimental session as the patient performed pointing motions in different contexts (see legend). Each point contains over 100 measurements from reaches to all targets and repetitions of the forward and backwards pointing loop. Each point is the maximum likelihood estimate of the shape and the scale parameters of the Gamma probability distribution, with 95% confidence intervals. The estimated values for a representative typical control are shown with a black star. Shifts to the right of the Gamma plane, toward typical regimes represent gains in predictability from trial to trial. Shifts down indicate reductions in noise. **(C)** Estimated mean and variance were obtained from the estimated shape and scale parameters of the Gamma probability distribution. The SD for each condition in the legend is plotted as a function of the mean for the patient in relation to the representative typical control. For each of the kinematics parameters of interest we also estimated the Gamma probability density function using the range of values of the parameters experimentally obtained for each condition. Left correspond to the time to peak velocity. Right correspond to the values of the peak velocity.

**FIGURE 6:**
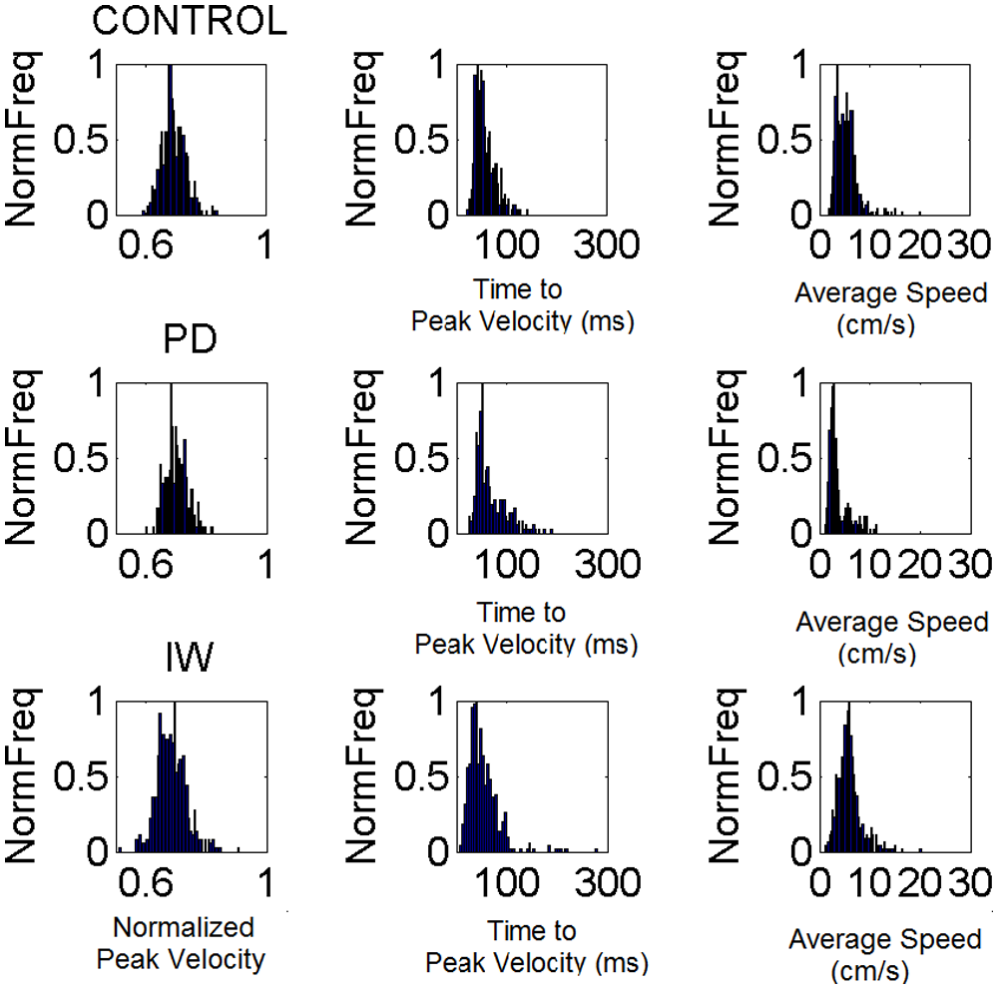
**Velocity-dependent frequency histograms from reaching data.** Data are from the visually guided motions of three representative subjects whose speed profiles are shown in **Figure 2**, (row 1 Control, row 2 PD, and row 3 Deafferented.) The frequency histograms of the normalized maximum velocity, the time to reach this peak velocity (ms) and the averaged trial speed (cm/s) are shown in each case.

In *Step 1* we gather the kinematic parameter of interest (e.g., the normalized peak velocity) for over a 100 repeats of the motion (see for example **Figure 5A** showing normalized frequency histograms from IW in different sensory guidance conditions. **Figure 6** shows normalized frequency histograms for the normalized peak velocity, the time to reach the peak and the averaged speed of the segment). The histograms and estimation of bin size for the parameters of interest were obtained using in-house developed Matlab routines based on well-established algorithms for optimal bin estimation with *W* = 3.49σ*N*^-1/_3_^ (Izenman, 1991) where *W* is the width of the bin, σ the SD of the distribution (we used estimated SD $\overset{\wedge }{\tau }$) and *N* is the number of samples. We then use maximum likelihood estimation (m.l.e.) to obtain with 95% confidence the estimates of the parameters of the probability distribution function best describing this random process over time. In a series of papers involving human subjects along the continuum of typical and atypical cases we have reported that the continuous Gamma family of probability distributions captures well the statistics of human motor output variability (Torres, 2011, 2013a,b). The Gamma probability distribution has probability density function given by:

(1) $$y=f(x\,\;|\;a,b)=\frac{1}{b^{a}\Gamma \left(a\right)}x^{a-1}e^{-\frac{x}{b}}\,$$

with shape (*a*) and scale (*b*) parameters and the Γ function. By varying the shape and scale parameters, one can move from a Gaussian-like distribution to the exponential distribution, with skewed distributions in between the two extremes. *Step 1* of **Figure 4** shows examples of frequency histograms of a typical and an atypical subject with the corresponding best fitting probability distribution function curves in red.

In *Step 2* of **Figure 4** we show different probability density function curves generated using (*a, b*) parameters experimentally estimated from various human movement data and experimental values for the ranges of the velocity-dependent parameters. This estimation step gives us a sense of the family of probability density functions describing the data well.

In *Step 3* of **Figure 4** we plot the estimated parameters for each person in Step 1 on the (*a, b*)-Gamma plane. We use the Gamma plane here to map the individual points representing each participant because we have found that this family captures well the statistical features and shifts of human motor output variability. This step enables localization of each person with 95% confidence on the Gamma plane. This step also enables classification of the person relative to a well known atypical case. We can use this map to establish, whenever possible, relationships between the manifestation of a disorder that is evaluated or detected by observation, and well understood causes of similar manifestations. In our specific case, we can then localize on the Gamma plane the stochastic signatures of the velocity dependent parameters estimated from the motor output variability of the subject without proprioception. Under similar experimental conditions, in IW these signatures would indicate the specific statistical patterns due to lack of proprioception, from not being able to sense the movements continuously. We can then classify other participants relative to this extreme signature and infer with some certainty that their motor output deficiencies may be also partly due to deficiencies in the sensing of movement (proprioceptive deficiency). Specifically, we propose that failure in systematically detecting subtle fluctuations in sensory inputs from continuous movements would prevent the formation of a reliable and predictable expected value. Notice here that the detecting capabilities of our sensory systems are not something that we are fully aware of. IW has trained to move guided by vision and has developed, over many years, compensatory strategies that mask his lack of proprioception quite well. Likewise, these participants with PD were able to improve their kinematic performance when guided by vision (Torres et al., 2011). Yet, these improvements were at the expense of having to monitor their motions very closely, using the same types of movements that would otherwise be highly habitual and automated in neurotypical controls.

In both IW and the participants with PD we can cast the data relative to the patterns obtained from the young controls performing similar motions. We can then learn about healthy patterns of motor output variability in forward and back reaches and quantify where they localize on the Gamma plane in reference to patterns of motion under total lack of proprioception (IW) or under noisy and random motor output variability systematically leading to corrupted kinesthetic reafference as PD severity increases.

The next *Step 4* enables tracking, in real time, the shifts in the stochastic signatures as a function of the type of external sensory input used to guide the movements. This allows us to extract the form of external guidance that leads the motor output variability toward statistical regimes of anticipatory behavior. Points up and to the left of the Gamma plane, near *a = 1* the particular extreme case of the exponential probability distribution, indicate a tendency toward random and noisy patterns. At the other extreme, points down and to the right of the Gamma plane indicate tendency toward reliable and predictable patterns. Randomness here means that along the continuous motions, prior events (e.g., the peak velocity values and timing of the motion in prior trials) do not contribute to the accumulation of an expected value indicative of those features in future events. Anticipatory patterns on the other hand mean that prior events tend to accumulate information toward a reliable expected value (Ross, 2009). In previous work, based on these principles we have derived velocity and acceleration dependent stochastic rules showing the predictability of natural motions (Torres, 2013b) but such rules fall out of the scope of this paper.

The statistical parameters of the Gamma probability distribution according to the estimated shape (*a*) and scale (*b*) are: *mean = a.b* and *var = a.b^2^*. These are used in the computation of the noise-to-signal ratio [the Fano Factor (Fano, 1947)] given by the variance divided by the mean. This ratio is *b* in the Gamma case. Therefore, the *b*-scale parameter of the Gamma probability distribution informs us of noise-to-signal levels. The values of both the shape and the scale parameters change over time for continuous behavior (Torres, 2013a; Torres et al., 2013a). They change as we adapt to new contexts, as we perform new movements and as our sensory-motor systems are impacted by external input (Torres, 2013b; Torres et al., 2013a,d). In **Figure 5B** we discuss this real-time tracking capability of these analytical tools and use IW as an example (as we did in other PD-related work (Torres, 2013a).

#### Statistical indexes

Once we localize each participant on the Gamma plane, we can obtain an index of similarity with respect to IW. To do this we compute the Euclidean distance from each (*a, b*) point on the Gamma plane to the deafferented subject’s location. This quantity is plotted in **Figure 9A** relative to young controls and **Figures 9A,B** for the mild PD patients relative to IW’s best case scenario (visual guidance.) We obtain the noise-to-signal levels given by the Fano Factor as a function of this distance. We can then identify relations between the subject’s absence of proprioception and the level of proprioceptive deficiency that the signatures of motor output variability reflect for each one of the patients with mild PD, severe PD and for the elderly controls.

## RESULTS

### THE SUBJECT WITHOUT PROPRIOCEPTION

The velocity-dependent parameters of the subject IW yielded frequency histograms that were well fit by the continuous Gamma family of probability distributions. The estimated shape and scale parameters with 95% confidence according to m.l.e. were non-stationary. They shifted their values as a function of the type of sensory guidance. They ranged from values close to the exponential distribution (shape *a* close to 1) in the dark or when different speeds and loads were used, to values in the more symmetric (Gaussian) range of the Gamma (*a, b*)-plane, close to the stochastic signatures of the representative normal control. The latter was the case under some form of visual sensory guidance, e.g., in a lit room or in a dark room with visualization of the target, or of the moving finger.

From trial to trial his memory guided reaches in the dark were noisier and more random than those under visual guidance. These results are plotted in **Figure 5**. This figure shows representative normalized frequency histograms of the time to reach the peak velocity and of the values of the peak velocity under different contexts. In the top plots of the **Figure 5A** we see the frequency histograms of these parameters under different dynamics (fast and slow instructed speeds and lifting small loads). In the bottom plots of the **Figure 5A** we see the normalized frequency histograms of these parameters when the reaches are under visual guidance of the target. These are comparable to those of the representative control. This can be appreciated in **Figure 5B** where we plot the different estimates for each condition. We also show it in **Figure 6** where we plot IW’s signatures and highlight the frequency histograms and their changes from the dark condition (black star) to the condition where he uses visual guidance (green star).

Notice here that the shifts in stochastic signatures (non-stationary statistics) were within time scales of minutes of the experimental session. Each motion segment took on average between 1 and 2.5 s depending on the subject type. For example, across 100 cycles of one experimental manipulation (e.g., spanning approximately 4–5 min of pointing motions with guidance of the pointing finger) we obtained the estimated parameters of the Gamma distribution. In the next 5 min of the experimental session with the next experimental manipulation (e.g., pointing at different speeds, or pointing this time with continuous vision of the target, etc.) the estimated parameters shift on the Gamma plane. Over the time scales of minutes we can then start building a stochastic trajectory and estimate the rate of change of the shifts.

The shifts in stochastic signatures are under the volitional control of the system performing the task. In line with von Holst’s principle of reafference (Von Holst and Mittelstaedt, 1950; Von Holst, 1954), the motor output at time *t* becomes kinesthetic sensory input at time *t*+*k* that the prior motions themselves caused. The value of *k* refers to a time elapsed between measurements that we researchers take according to the sampling resolution of our sensors. For the nervous system, this time will depend on transduction and transmission delays of the sensors involved. The accumulation of this information over time under optimal guiding conditions (e.g., visual guidance in IW) lead to systematic shifts of signatures on the Gamma plane corresponding to reliable expectations toward the Gaussian regimes of behavior in the best cases or to the exponential (random and memoryless) regime in the worst cases.

Because these changes in motion signatures are produced by the system itself, any prediction (and later confirmation) by the system is based on its own non-stationary statistics. Any anticipation of future kinesthetic sensory consequences of the ongoing action is directly tied to prior motor information that the system itself caused. In other words, in this continuous kinesthetic reafferent loop, under good kinesthetic guidance, prior events reliably contribute to the estimation of future events. Under bad kinesthetic guidance, this is not the case. We show this with the corresponding probability distributions, *empirically estimated from the continuous data*. We continuously sample from this loop and provide analytics to estimate the shifts of the estimated parameters within time scales that are relevant to the experimental session.

Notice also how the stochastic trajectory of the subject without proprioception approaches the normal elderly control values in both velocity-dependent parameters (value and timing) when he closes the feedback loop using visual guidance. In this case, the parameters from the elderly control are obtained from similar visually guided conditions as those of the subject without proprioception. In **Figure 5B** we take the signatures of IW across the Gamma plane from highly reliable statistical regime under visual guidance (magenta) to extremely random and noisy (red), to less random and less noisier (blue) in the dark, to very reliable and anticipatory regime again while using visual guidance in full light. We shift his motions toward patterns of the typical control (black star; or away from these typical regimes (red dots)] in a systematic way that we can control using these methods.

**Figure 5C** shows the estimated mean and variance parameters for each condition obtained with the estimated shape and scale parameters of the Gamma probability distribution. The patterns of variability of the subject are also affected by the type of sensory guidance and context. Visual guidance and a lit room make his motions faster and less variable. His speed is fastest for the condition when he has visual guidance. Under visual guidance of either the target or the moving finger in the dark, he moves the arm in feedforward mode, reaches peak velocity at 430 ms on average. He then decelerates to the target with multiple peaks, faster on average than the control (500 ms on average), and with speed variability in the normal range. When the room is lit, he also reaches peak velocity around 450 ms on average, close to the representative control. Under this condition (which is how he often moves in natural circumstances), he has comparable speed values and variability to those of the controls. For the conditions where he is in the dark or where the dynamics of the motion were manipulated, he differs from the controls significantly (Kruskal–Wallis non parametric ANOVA, *p* < 0.01). He reaches peak velocity significantly later than the controls and has higher variability (**Figure 5C**). His peak velocity is faster in the dark than under different instructed speeds or loads. In the latter cases, his variability and the noise-to-signal (Fano Factor) are at the highest for both parameters. These systematic changes can also be appreciated in relation to the representative age-matched control in the plotting the Gamma probability density functions experimentally estimated.

### POWER LAW CAPTURES SYSTEMATIC TRENDS BETWEEN PD SEVERITY AND THE SUBJECT WITHOUT PROPRIOCEPTION

**FIGURE 7:**
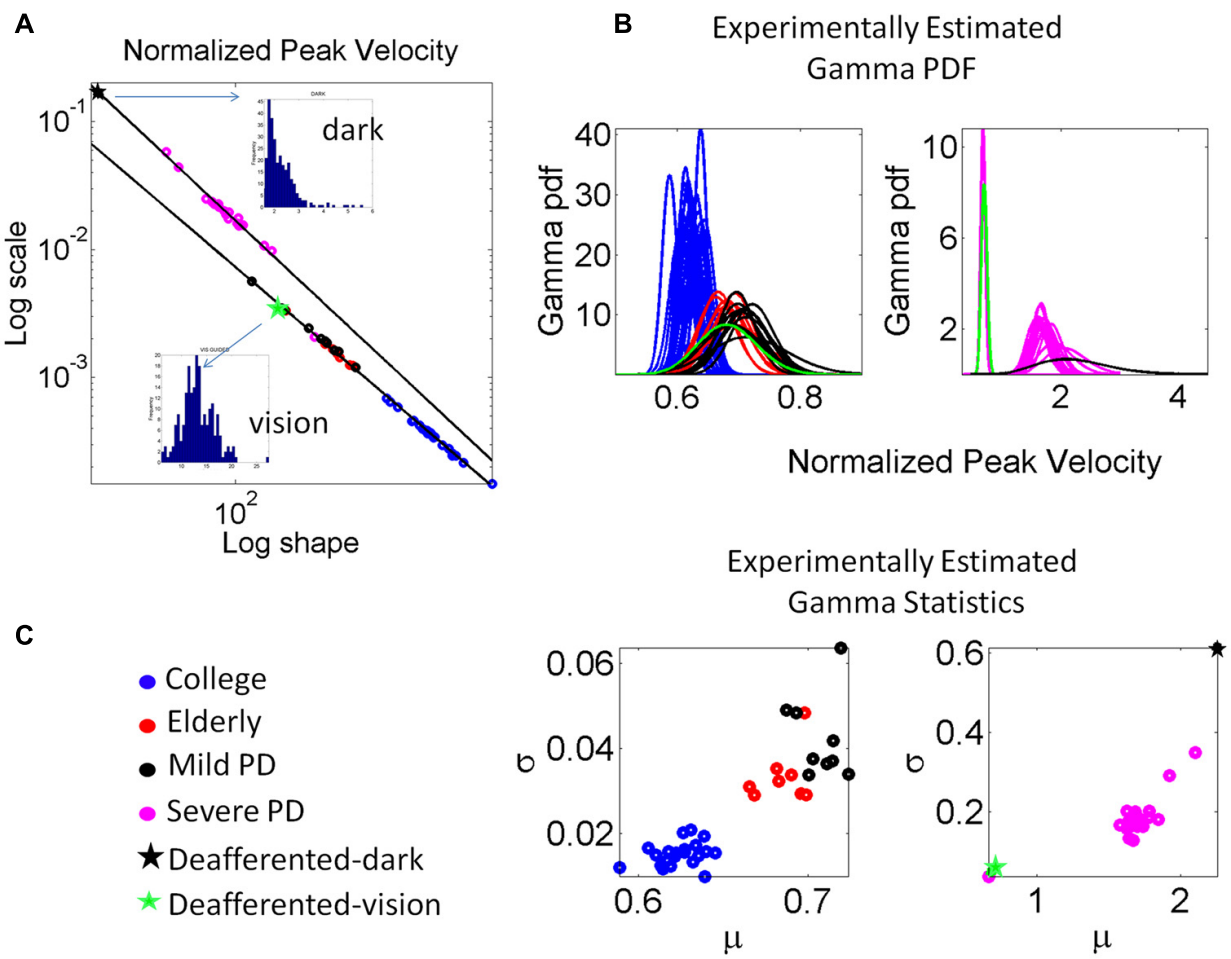
**Estimation and classification process. (A)** The log-log plot of the Gamma plane shows the spread of the cohort including college students, elderly participants approximately matching the age of the PD patients, the patients in the mild, and more advance stages of PD and the deafferented subject. The deafferented subject’s data correspond to pointing motions in complete darkness (black star) and motions under visual guidance (green star). Each point is estimated with 95% confidence and represents the signature of the velocity-dependent motor output variability, specifically obtained from the normalized peak velocity. Notice the change in the slope and intercepts for the power laws revealed by the data (see details in the text.) Frequency histograms of the peak velocities of the dark and visually guided conditions for the deafferented subject are shown as insets in **(A)**. **(B)** The estimated Gamma probability density function curves are obtained using the estimated Gamma shape and scale parameters and the experimentally determined range of the normalized peak velocity. Each curve corresponds to the point in **(A)**. Left shows the mild PD group in relation to elderly and young controls. Right shows the group with more advanced PD using as anchors the data from the deafferented patient while pointing in the dark and with visual guidance. **(C)** Estimated mean and variance to obtain the SD and automatically classify the participants. Notice that higher values of μ correspond to lower values of averaged hand speed in the denominator term of the normalized peak velocity index (see text for the definition of normalized peak velocity.)

We examined the PD patients with different levels of severity using these analytics. We also examined the deafferented subject, the age matched controls and the young controls under different scenarios. **Figure 6** shows representative normalized frequency histograms of the normalized peak velocity, the time to reach the peak velocity and the average trial speed. **Figure 7** reveals the location on the Gamma plane of the mild PD patients and of the patients with advanced PD relative to the deafferented subject and to the controls. The deafferented subject appears twice on the Gamma plane. One location corresponds to his signatures under visual guidance (green star) closer to the mild PD patients. The other location corresponds to his signatures in the dark (black star) closer to the severe PD patients, except for one patient in the severe PD group who falls with the mild PD group closer to IW under vision. The trends were systematic and captured by a power law with distinct exponents (slopes) and intercepts for mild and severe PD in relation to the deafferented subject’s signatures with vision and in the dark, respectively.

The scatter corresponding to the mild PD patients and the deafferented subject under visual guidance were well fit by an exponential fit to this relation with f (x) = αe^βx^ where *f(x)* is the scale parameter (*b*) and *x* is the shape parameter (*a*) on the Gamma plane. The coefficients with 95% confidence bounds were α = 0.9066 [0.8423,0.9709] and β = -1.047 [-1.06, -1.034]. The goodness of fit parameters was: Sum Squared Error (SSE), 5.37 × 10^-8^, R^2^, 0.99, and Root Mean Squared Error (RMSE), 3.81 × 10^-5^.

The scatter corresponding to the severe PD patients and the deafferented subject in the dark shifted on the Gamma plane. They were well fit by an exponential fit as well with α = 3.315 [3.079, 3.551] and β = -1.147 [-1.17, -1.124]. The goodness of fit parameters was: SSE, 4.11 × 10^-5^, R^2^, 0.99, and RMSE, 0.001512.

Under visual guidance mild PD patients tend toward the deafferented patient’s signatures and away from the young controls. Most elderly controls separate from the mild PD patients. This can be appreciated in **Figure 7C** showing the estimated mean and variance of the empirically estimated Gamma probability distribution. Notice here that as PD severity increases the signatures shift places on the Gamma plane and approach those of the deafferented subject in the dark. Insets in the **Figure 7A** contrast the frequency histograms of the velocity peaks of the deafferented subject (ranging in the dark from 1 to 5.5 cm/s and increasing with vision in values and range, 5–25 cm/s). **Figure 7B** shows the probability density functions estimated from the empirical data. Notice here that the deafferented subject (green trace) has very high variability and very low speed on average. This trend is also systematically observed in the PD patients. The mild PD are slower than the deafferented subject during the visual guidance condition, but much more variable and slower than the young controls. The severe PD patients in **Figure 7A** fall between the deafferented subject under visual guidance (green star) and the deafferented subject in the dark (black star). In **Figure 7B** we plot the probability density functions (pdf’s) of all subjects under visual guidance in relation to IW using vision (green pdf curve on the left) and IW in the dark (black pdf curve on the right). **Figure 7C** shows this information as well. Notice the outlier patient in the severe group who is comparable to IW under visual guidance (next to the green star) but worse than the mild PD patients (two subplots are used because of the increase in noise levels along the vertical axes for severe PD and IW in the dark.)

In general the PD patients move systematically slower, thus resulting in higher values of the normalized peak velocity (average speed in the denominator is lower in value than for normal controls). Likewise the variability systematically increased with the severity of the PD patients. The mild PD remained systematically closer to the subject without proprioception in the visually guided condition. The severe PD were systematically closer to the deafferented subject in the dark condition. The elderly controls also separated on the Gamma plane of **Figure 7A** from the young college students. This can also be seen in **Figure 7C** in the slower (on average) and noisier motions of the elderly controls compared to those of the young controls. (Here recall that lower values of the normalized peak velocity to the left of the horizontal μ-axis are due to the higher average speed trial.) The trial-to-trial motor variability of the elderly was very high in general, but in particular this was the case also for those with mild PD. The severe PD patients were in a different scale altogether and had to be shown in different plots to the right of **Figures 7B and 6**. Notice that their estimated Gamma statistics fall between those of the deafferented subject under visual guidance (best case scenario) and in the dark (worst case scenario.)

### FORWARD vs. RETRACTING PATTERNS SEGREGATE SEVERITY OF PD PATIENTS

**FIGURE 8:**
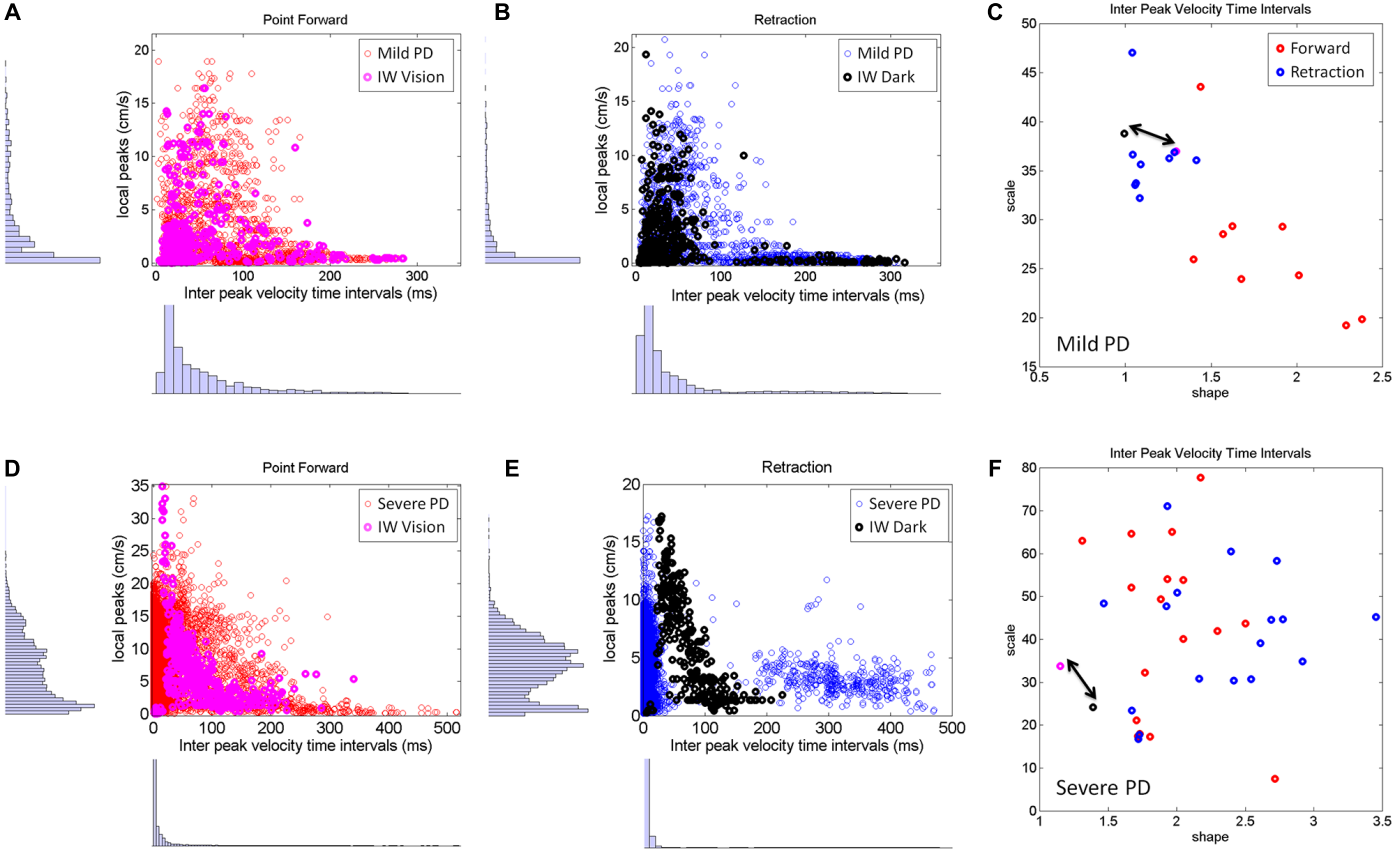
**Differences between mild and severe PD patients in the inter-time intervals of local velocity peaks. (A–C)** Statistical features of temporal dynamics from mild PD patients’ forward pointing motions and retractions in relation to IW in the visually guided condition. Scatters of all local velocity peaks as a function of the inter time intervals between local peaks with frequency histograms corresponding to the values of these local peaks (y-axis) and their inter time intervals (x-axis). **(C)** The Gamma plane scatter of the mild PD patients’ signatures of the inter time intervals of the local velocity peaks. Notice that points corresponding to the retractions are in the noisier and more random region of the Gamma plane where IW’s (both forward and retracting) motions fall (marked by the double arrow). The points corresponding to the forward pointing motions separate from those corresponding to the retractions in all (but one) mild PD patients. **(D–F)** Similar plots as **(A,B)** corresponding to severe PD patients in relation to IW in the dark condition. Note the difference in scatter and multimodality of the y-axis frequency histograms (see text for additional details). **(F)** The scatter of points of the severe PD patients in the Gamma plane do not distinguish between forward and retracting motions. Notice that the majority of the patients have noisier patterns of inter peak time intervals than IW. Notice as well that IW patterns are at the exponential end (random and memoryless probability distribution) of the Gamma plane.

The visible differences between the forward and retracting speed profiles across PD patients observed in **Figure 2** were further studied. The patterns of additional local peaks and their inter-time intervals are shown in **Figures 8A,B** for the mild PD patients in the forward (**Figure 8A**) and retraction segments (**Figure 8B**). **Figure 8C** shows a distinct separation between the signatures of the inter-time intervals of the local peaks. The local peaks reflect action tremor that varies from patient to patient. Here the temporal dynamics of those local peaks indicates that the retractions are under a different level of noise and randomness than the forward motions in the mild PD group. Furthermore, the retraction signatures are similar to the signatures of IW under visual guidance, signaled in **C** by a double arrow. IW signatures for both forward and retracting motions are in the random, memoryless, and noisy regimes of the Gamma plane. The level of noise (y-axis values) fall for most of the PD patients below IW’s levels.

The second group of PD patients (**Figure 8**, bottom row) showed several distinct patterns worthwhile discussing. The first was the presence of multiple modes in their individual frequency histograms. We performed the Hartigan’s dip test of unimodality (Hartigan and Hartigan, 1985) and found significant differences in this group of PD patients, 11/17 in the forward case and 14/17 in the retraction (for *p* < 0.05). These differences were not as marked in the mild group of patients (**Figures 8A–C**). This was the case both for the values of the local peaks and for the frequency histograms of their inter-time intervals.

As an ensemble the differences in multimodality can also be seen in **Figures 8D,E**, as compared to **Figures 8A,B**. Yet, the results from the individualized analysis were more striking. The results from the analyses of the Gamma plane in **Figure 8F** are in reference to the first mode of the individual distributions, the mode comprising a range of values comparable to those in the patients of **Figure 8C**. Their stochastic signatures from the inter time intervals of the local peaks revealed a far more mixed picture between the forward and retraction segments. The patterns from the dark condition for subject IW are marked by a double arrow. His signatures stand at the left end of the Gamma plane (random and memoryless exponential regime.) They are noisy as well (high values of the Fano Factor which is the scale value on the y-axis.) Yet, notice that those signatures of the temporal dynamics of the local peaks in PD patients are even noisier than IW’s in the dark.

In summary, the temporal dynamics of the action tremor of these severe PD patients turned out to differ from those of the other PD group: (1) Their overall frequency histograms had several modes; (2) The mode corresponding to the range of values of the (**Figure 8D**) could not be separated into forward and retraction segments in the Gamma plane; (3) These patterns on the Gamma plane were for most of those PD patients noisier than IW’s [in contrast to those in (**Figure 8C**)]. Lastly IW’s inter-time intervals for these local peaks were noisier with vision than in the dark condition (where he may have used motor imagery) and just as random, memoryless, and unpredictable in both cases. The noisier patterns in the case of visual guidance may be partly contributed to by vision itself, but we have no way at present to know this with any degree of certainty.

### OTHER (PROPOSED) INDEXES OF SEVERITY

**FIGURE 9:**
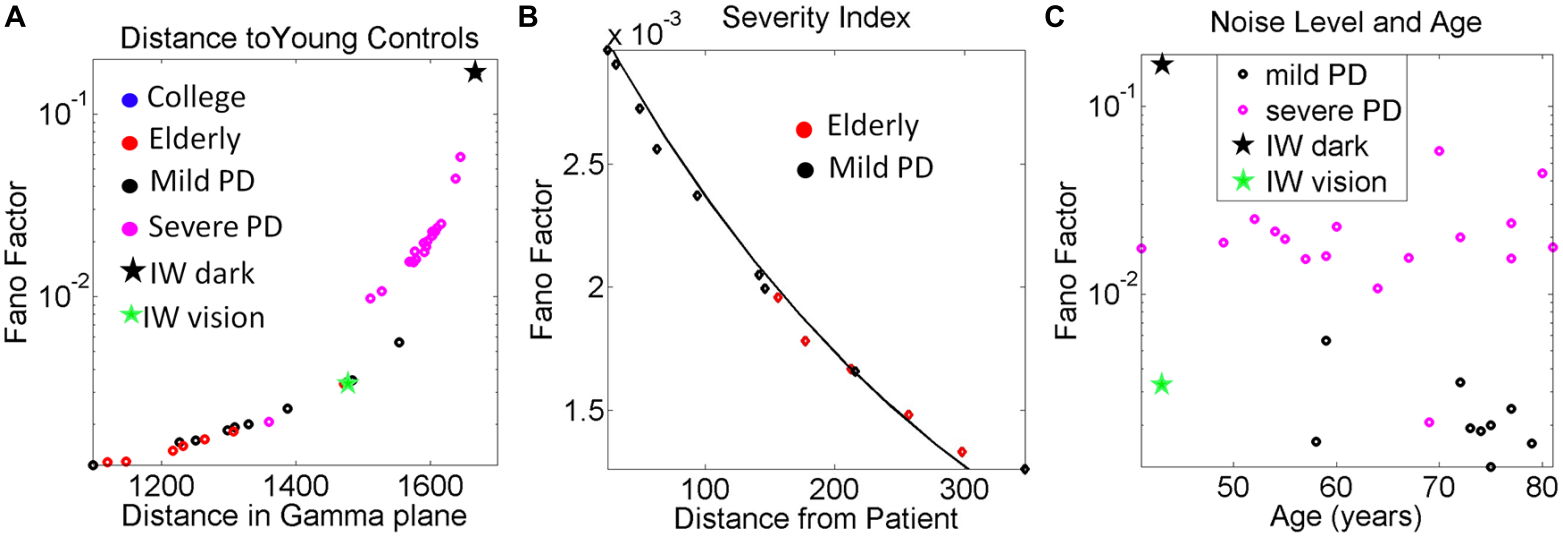
**Other variability dependent indexes to quantify the relations between motor output variability and proprioceptive deficits. (A)** Systematically measuring the distance in the Gamma plane between each participant and the centroid location of the young control group reveals an exponential increase in the noise levels (measured by the Fano Factor, the ratio of estimated variance over estimated mean) as the participant is farther apart from the young controls’ centroid. **(B)** Zooming in the mild PD provides a putative index of severity even at that mild stage of PD to quantify decrease in kinesthetic sensing relative to the deafferented subject according to noise levels in the motor output variability. The exponential relation of this segment has a shift in slope and intercept with respect to the severe PD group (see text for details and **Figure 6**). In both groups the closer the stochastic signature of the velocity-dependent motor output variability of the person is to the deafferented subject, the noisier the signal of that person is (higher Fano Factor value). **(C)** Noise to signal levels (Fano Factor) as a function of age in Mild PD vs. severe PD patients.

The 3D kinematic data from natural unconstrained behaviors is very rich in information. The layer of estimated stochastic signatures that we extract from the raw kinematics can be used to build many indexes that can inform us of the noise-signal flows in the compromised systems and in the normally aging systems. One such index is shown in **Figure 9A**. We obtained for each participant the Fano Factor (estimated variance/estimated mean) as a function of the Euclidean distance in the Gamma plane from each point to the location of the centroid of the young controls’ scatter. The closer to the young controls the person was, the lower the noise. In **Figure 9B** we zoomed in for the mild PD patients and show their distance from the deafferented subject under visual guidance. The closer the person is to the deafferented subject, the higher the noise level in the motor output variability. We found an exponential fit to this relation with *f* (x) = α*e*^βx^ where *f(x)* is the Fano Factor and *x* is the Euclidean distance from the participant IW to a mild PD subject on the Gamma plane.

The coefficients with 95% confidence bounds were α = 0.0033 [0.0032, 0.0034] and β = -0.0031 [-0.0033, -0.0029]. The goodness of fit parameters was: SSE 1.86 × 10^-7^, R^2^ 0.983, and RMSE 1.04 × 10^-4^.

The plot comparing the levels of the noise to signal ratio (the Fano Factor) in the mild vs. severe groups of patients as a function of age is shown in **Figure 9C**. The plot revealed a trend that manifested regardless of age. The noise levels were higher in the severe PD group than in the mild PD group. All participants but one in the severe PD group had values of the Fano Factor above those in the mild PD group. The latter had noise levels comparable to those of IW under visual guidance. In the dark, the noise levels of the patterns of IW were higher than those of the severe PD patients.

## DISCUSSION

This work characterized the stochastic patterns of motor output variability of a subject without proprioception under different pointing conditions. His stochastic patterns were compared to those obtained from patients with various degrees of PD severity and to age-matched normal controls. They were also compared to those of college undergraduate students who performed similar 3D pointing motions in a lit room. We focused on the patterns of velocity dependent variability as the movements continuously unfolded in 3D, rather than on discrete trials with the endpoint spread at the target. Our hypothesis was that the patients with PD would have velocity dependent patterns of variability closer to those of the subject without proprioception than to the healthy aging controls. We searched for evidence in support of the notion that *deficiencies in kinesthesia may emerge, even at a mild stage of PD, when statistically noisy and random motor output variability persists over years*. We specifically refer to kinesthesia as the stable and reliable detection (e.g., by mechanoreceptors) of continuous body motions. This detection capacity we presume emerges from the sensation of continuous body and limb movements. We examined this hypothesis using novel statistical techniques that treat minute fluctuations in the velocity dependent motor output as a continuous random process.

The new statistical methodology that we used is centered on individual assessments of the continuous flow of movements (Torres and Jose, 2012). In this context we are not aiming at correlating movement parameters with treatments of the data. Rather, we are empirically estimating the underlying probability distributions that most likely characterize the continuously returning afferent stream that those motions themselves caused. In other words, we provide a novel statistical characterization of von Holst’s concept of kinesthetic reafference.

We examined the minute velocity-dependent fluctuations that are inherently present in the continuous flow of pointing motions. We estimated the statistical parameters of the continuous Gamma family of probability distributions and showed how these parameters directly estimated from the physical motions of the finger, shifted systematically with manipulations of the context and the form of sensory guidance. We showed self emerging systematic patterns in mild and severe PD patients in relation to the deafferented subject, under different forms of sensory guidance.

### SYSTEMATIC TRENDS TOWARD THE STOCHASTIC SIGNATURES OF IW

We found consistently corrupted patterns of motor output variability in subjects with PD that shifted toward the signatures of the deafferented patient revealing different degrees of PD severity. The trial by trial motor-fluctuation patterns of these patients increased the noise levels with a consistent tendency approaching the patterns of IW in the absence of vision. These trends were captured by a power law that shifted slope and intercept with PD severity levels.

The rate of change of the progression of this disorder is rather unique to each person and depends on many factors; including age, age of onset, genetics, and environmental factors, among others. Our new statistical platform permits a type of individualized analyses where the person is his/her own control. We can measure the shifts in the stochastic signatures of each person in one condition relative to another condition, with respect the baseline signatures of that person within a given context. Any similarity among subjects under a given condition reveals self emerging clusters that can be indicative of a group behavior. In this study the PD patients automatically revealed different groupings in the Gamma plane.

The striking differences in statistical classes between clusters resulting from the comparison between forward and retracting segments further supported systematic trends in severity. The systematicity of this trend was confirmed through the analyses of both the global peak velocities of the segments and at an even finer level, by the stochastic signatures of their local peaks and their inter-time intervals. Mild PD patients had better control of the forward than of the retracting segments. The latter resembled the patterns of IW with visual guidance. In contrast, severe PD patients showed that the patterns from both segments were indistinguishable and comparable to IW’s patterns without vision. These trends were also found in the Fano Factor (the noise to signal ratio) which in all severe PD patients (but one) was higher than in the mild PD group. This result was independent of the age of the patient. For example, we found older patients with mild PD with lower levels of noise than younger patients with severe PD. This suggests that the levels of noise present in the velocity dependent spatio-temporal parameters may be a good predictor of severity level in PD. That result confirms similar findings in acceleration patterns of general everyday motions in PD (Torres, 2013a).

We propose that, given the systematic trends found here in the temporal dynamics, and noise levels of the global and local velocity peaks, there may a disruption in central control inherent to PD. This, we propose may be partly due to the uncertainties from reduced motor stability which within the closed-loop formulation of our problem, would also result in uncertainties in corollary discharge impeding strategies for predictive forward control.

In subject IW we know the source of the problem. Yet in the PD patients we do not know where the problem originates. The persistent corrupted motor output with continuously noisy and random signatures of narrow bandwidth could impede a number of processes required for proper sensory motor integration, estimation, and prediction. As in any data-driven study, one could only speculate on the source of the problem and further test several hypotheses in future studies. Here are some of these guesses: Perhaps the corrupted motor output poses a detections problem for the kinesthetic mechanoreceptors sensing the continuous flow of motion. Change detection along this flow would be compromised, as receptors would be continuously sensing random and noisy events, thus impeding the formation of a systematic and reliable expected value that the system could predict. The exponential distribution characterizing their fluctuations in motor output is the most random (“memoryless”) distribution. Previous events do not contribute to future events any more than current events do. Perhaps this lack of stability would also transfer to the transmission of the signal throughout the sensory nerves. Even if they were not physically damaged in PD (as in the case of IW) the corrupted, feedback with narrow bandwidth would affect the transmission and transduction process. Perhaps the process of integration of this form of motor-based sensory feedback with other sensory signals is impeded and at the central level estimation and prediction are also impeded. All these factors could contribute to the disruption in corollary discharge. Systematic testing in our labs is warranted in future PD studies to address each level of the above mentioned hypotheses.

IW had velocity-dependent reaching patterns of motor output variability that were well characterized by the continuous Gamma family of probability distributions. The estimated shape and scale parameters of this family of distributions shifted systematically in this subject as a function of the form of sensory guidance that we provided within the experimental session. The estimated Gamma distribution parameters spanned values from the exponential range (the shape parameter near 1) for movements made in the dark or with manipulations of dynamics, to skewed ranges closer to those of typical controls, tending toward the symmetric Gaussian (shape values above 10) for movements with visual guidance. These shifts in the subject had different rates that were systematically driven as a function of the form of sensory guidance that we provided during the experiments. When we provided visual feedback, we systematically drove his stochastic signatures down and to the right of the Gamma plane, i.e., the noise levels of the velocity-dependent motor output fluctuations dampened along the scale axis and the reliability and predictability of their expected values increased along the shape axis. When we turned off the lights and/or manipulated the dynamics, we systematically drove his stochastic signatures up and to the left of the Gamma plane, i.e., toward noisy and random statistical regimes of the motor output fluctuations.

These non-stationary features of the statistics of continuously flowing motions were captured even within the time scales of the experimental session. This result paired with the fact that the probability distributions of these velocity-dependent parameters were non Gaussian strongly suggest that we should not take averages of hand kinematic parameters and/or assume Gaussian priors when assessing sensory-motor variability in general. They also point to the potential use of these new methods in the design of individualized therapies tailored to exploit the predispositions and best sensory capabilities of the person. Those predispositions can be extracted from the conditions leading to shifts on the Gamma plane that bring the patterns down and to the right, toward typical reliable and anticipatory statistical regimes.

In particular, the new results show that, without vision, the patterns of motor output variability of the deafferented subject were random and noisy. They fell toward the exponential range of the Gamma (*shape, scale*)-plane with shape values close to 1. Under this “memoryless” distribution every trial is like a new trial. This feature would require feed-forward strategies predicting ahead sensory consequences (movements in neurotypical cases) of impending actions, guided by vision in IW’s case (Balslev et al., 2007; Miall and Cole, 2007). Without vision, his moment-to-moment movement output variability fails to provide a stable enough motor percept (a motor prior) to rely on under new contexts.

When guided by vision IW’s average speed was comparable to that of age-matched normal controls. Yet, his signature of variability was closer to those of the patients with mild PD than to those of the age-matched controls. In the dark, his patterns were closer to the severe PD patients. This suggests that the systematically noisier and more random motor output variability quantified in these PD patients as their severity levels increased may relate to impairments in kinesthetic reafferent information during continuous motions.

The exponential trend in the stochastic signatures of motor output fluctuations found here in subjects with mild and severe PD for this pointing task indicates reliance in the “here and now,” with no cumulative information from past motor events contributing to the estimation, prediction and confirmation of future motor events. This type of continuously random feedback may play a role in the lack of anticipatory control reported in other more complex tasks of the reaching family (Santello et al., 2004; Muratori et al., 2008; Yanovich et al., 2013).

It is important to bear in mind that IW’s loss of proprioception occurred nearly 25 years prior to our testing him, and that he underwent extensive rehabilitation, and unlike other deafferented patients, learned to successfully walk unassisted as long as he had vision (Cole, 1995). His case shows us the limits of what is possible to achieve after extensive rehabilitation and reorganization following deafferentation in young adulthood (Cole, 1995). Perhaps if we had tested IW soon after his deafferentation, his data would have been much farther separated from those of these PD patients, who also have an over reliance in visual guidance. We are not implying that PD patients have proprioceptive deficits comparable to those seen following actual physical deafferentation. Rather than having damaged sensory nerves, as does IW, we here conjecture that the parkinsonian disorder may also involve impairments in the central processing of proprioception (Boraud et al., 2000; Rodriguez-Oroz et al., 2001; Escola et al., 2002; Seiss et al., 2003; Pessiglione et al., 2005). This may contribute to the persistence of corrupted motor output as a returning stream with narrowed bandwidth. The corrupted signal may be missed by the kinesthetic sensor’s radar at the periphery. In turn this would fail to form proper expected values for statistical estimation by the central controller. These are potential (presumed) mechanisms that we plan to explore in our future work.

In the introduction we proposed to term this putative deficiency “virtual deafferentation” to distinguish it from actual physical deafferentation of IW. If the sensory input from movement is continuously random and noisy, it is very unlikely that it can be used to construct an emergent, stable motor percept. Thus, sustained corrupted kinesthetic reafferent integration could potentially create a vicious cycle of sensory-motor dysfunction. We propose that the persistence of this cycle over time would systematically increase noise levels in both the motor output variability and the returning (reafferent) input stream. In this sense, these data both provide novel evidence for putative proprioceptive integration deficits in PD, and also show the extent of recovery humanly possible following deafferentation as an adult. IW’s systematic grouping with PD patients under his best to worst performance, as severity of the disorder increased in **Figure 7** supports both points.

The critical difference between the patterns of IW and those from most subjects with PD was the average speed of their reaches (**Figure 7C**). Despite the noise and randomness in his patterns, under visual guidance IW reaches had comparable mean values of the normalized speed index to those of the controls. This was in stark contrast to his performance in the dark. Across these conditions, the severe PD patients were systematically slower than the mild PD ones.

### POSSIBLE FUTURE APPLICATIONS OF THIS PARADIGM AND ANALYTICAL METHODS

The question of whether the source of noise at the periphery is due to deficiencies in detection by kinesthetic receptors, or physical sensory nerve damage, or central-level processing, or whether it is present at all levels, is an important one. We can further investigate this question using these new metrics across multiple populations of patients with known physical sensory nerve damage vs. patients without physical nerve damage but with corrupted returning stream of motor output. In this cohort we rather focused on the subject IW who we know had sensory nerve damage due to a viral infection (Cole, 1995, 1998; Cole and Paillard, 1995) and no recovery from it. We used his stochastic patterns in the dark as an anchor at one end of the Gamma plane (noisy and random patterns), and the patterns of young healthy participants at the other end (reliable and predictive patterns) to set bounds of worst and best case scenarios respectively, for the stochastic signatures of the PD patients. In future studies these new statistical methods could provide a starting point to further try and disentangle the contributions of peripheral sensory and motor signals to central control.

Lack of proprioception in other patients with neurological problems may be characterized by applying our methods to the motor output variability in relation to well known deafferented subjects like IW. Such severe purely sensory neuropathies can be seen in some cancer patients who receive chemotherapy, in Sjogren’s Syndrome and in the acute neuronopathy syndrome, as well as in some genetic disorders. The Gamma plane may serve as a map to localize the stochastic motor output patterns of PD patients (and of other patients) in relation to those obtained from the motions of well known patients whose proprioceptive impairments (as those of IW) are of known origins. Patients suffering from PD could also be localized relative to normal controls. This feature enabled us to assess shifts in patterns induced by specific forms of sensory guidance that tended to bring the subject’s performance toward normal ranges. Likewise, using IW and the normal controls as points of reference in the Gamma plane, it may be possible to know which forms of sensory guidance are most likely detrimental to the cohort under study. That would be the form of sensory guidance that systematically pulls their stochastic signatures away from normalcy and toward the patterns of the deafferented subject. Using the young healthy controls as the anchor for perfect cases also helps us track the form of sensory guidance that pulls the signatures toward the ideal healthy extreme on the Gamma plane.

It may be important to note here that the framework that we are using is different from the notion of internal models discussed in other work related to predictive vs. reactive central control strategies during grip force behavior (Nowak and Hermsdorfer, 2005). Under that framework the system has an internal model with a pre-programmed expected solution that is assumed to guide the error-correction process. In some contexts this information has been coined sensory motor priors when casting the problem using Bayesian statistics. Here the statistical framework that we use speaks of a continuous random process and its underlying emerging probability distributions, which we show have non-stationary parameters. At the core of our estimation process is the accumulation of evidence according to the continuous returning stream from the unfolding motions – both the motions that the person is volitionally controlling and those which the person is largely unaware of. At the core of their framework are contact forces that require compliance estimation and the assumption that an internal model must exist about the object dynamics and the internal dynamics of the system. Our pointing experiment does not require contact forces and our statistical platform do not assume any underlying sensory motor priors. It rather estimates what those probability distributions most likely may be under the contexts and situations that the experiment took place.

In recent years we have successfully used this new statistical platform to characterize the motor learning and adaptive behavior in athletes (Torres, 2011, 2013b), to characterize the autistic phenotype of known (Torres et al., 2013c) and unknown (Torres et al., 2013a) etiology, to identify gender differences in autism (Torres et al., 2013b), and to evoke volitional control in non-verbal so called “low functioning” children with autism (Torres et al., 2013d). We had also used it to track daily motion patterns in PD patients (Torres, 2013a). In the clinical cases, however, atypical the patterns of *motor output* variability were across these patient groups, we had no anchor to discern whether their noisy and random patterns were exclusively due to impairments in efferent output, or if there were also contributions from afferent deficits, particularly in relation to movement reafference and sensory-motor integration. The work with the deafferented subject IW, who lacks prioprioception from the neck down, strongly suggests that those velocity-dependent motor output fluctuations patterns with exponential signatures and high noise levels that we had previously identified are not only due to efferent noise. They may also relate to impairments in proprioception, specifically related to kinesthetic feedback integration and central control. Ongoing work in our laboratory is specifically mapping several patient types in relation to the deafferented subject IW.

## CONCLUSION

We have presented here a new statistical methodology that permits the characterization of motor output variability from the continuous flow of naturalistic behaviors. This methodology treats motor output noise-signal flows as a returning stream of sensory input that those motions themselves caused. The methods derive specific indexes to objectively and continuously quantify the evolution of such signatures over relatively short time scales. These time scales, relevant to a visit in the clinic, could enable tracking in real-time the shifts in stochastic signatures as a function of various forms of sensory guidance. Those shifts in the stochastic trajectories toward predictable and reliable statistical signatures would then signal which form of sensory guidance would be most adequate to treat the patient. Likewise, changes toward the random exponential regimes with higher noise-to-signal ratio would be identified as detrimental to the patient. In this sense the plasticity that we quantified here non-invasively in the motions from the peripheral limbs provide proof of concept that we can systematically induce and track changes under different forms of guidance. We could use such techniques in other patient populations besides PD. This work may enable the design of therapeutic interventions specifically targeting the patient’s sensory-motor capabilities and predispositions to reshape such motor output variability patterns toward normal statistical regimes. Such patterns of motor output variability integrated with external sensory guidance could provide the means to implement a new form of sensory-motor substitution or augment the sensory space of the individual, thus having broad therapeutic implications to induce and track adaptable changes in levels of volitional control.

## Conflict of Interest Statement

The authors declare that the research was conducted in the absence of any commercial or financial relationships that could be construed as a potential conflict of interest.
